# Sudden Cardiac Death Prevention in Patients with Ischemic Heart Disease—Beyond the Ejection Fraction

**DOI:** 10.31083/j.rcm2312409

**Published:** 2022-12-14

**Authors:** Hillel Steiner, Itzhak Sharabi, Ilan Goldenberg

**Affiliations:** ^1^Department of Cardiology, The Edith Wolfson Medical Center, 5822012 Holon, Israel; Sackler School of Medicine, Tel Aviv University, 6997801 Tel Aviv, Israel; ^2^The Adelson School of Health, Ariel University, 4076414 Ariel, Israel; ^3^Clinical Cardiovascular Research Center, Cardiology Division, Department of Medicine, University of Rochester Medical Center, Rochester, NY 14642, USA

**Keywords:** sudden cardiac death, ischemic heart disease, risk stratification, electrocardiography, biomarkers, echocardiography, programmed ventricular stimulation, cardiac magnetic resonance imaging

## Abstract

Sudden cardiac death (SCD) in patients with ischemic heart disease remains a leading cause of death. Prediction of who is at risk is based on the left ventricular ejection fraction (EF). However, the majority of victims of SCD have a normal EF, and the majority of patients implanted with an implantable cardioverter- defibrillator based on their EF are never treated by their device. Several parameters could allow better prediction of SCD. Several signs on the ECG and Periodic Repolarization Dynamics have been associated with increased risk. Elevated serum biomarkers such as pro-B type natriuretic peptides and serum soluble suppression of tumorigenicity 2 protein (sST2) are predictive of SCD. On the echocardiogram, global longitudinal strain, speckle tracking and relative wall thickness have been implicated. Programmed ventricular stimulation studies and cardiac magnetic resonance are promising modalities that could be further investigated. In conclusion, the EF is an imperfect tool for predicting SCD. Using the modalities reviewed, a model could be created for better prediction of patients at risk.

## 1. Introduction 

**Fig. 1.**
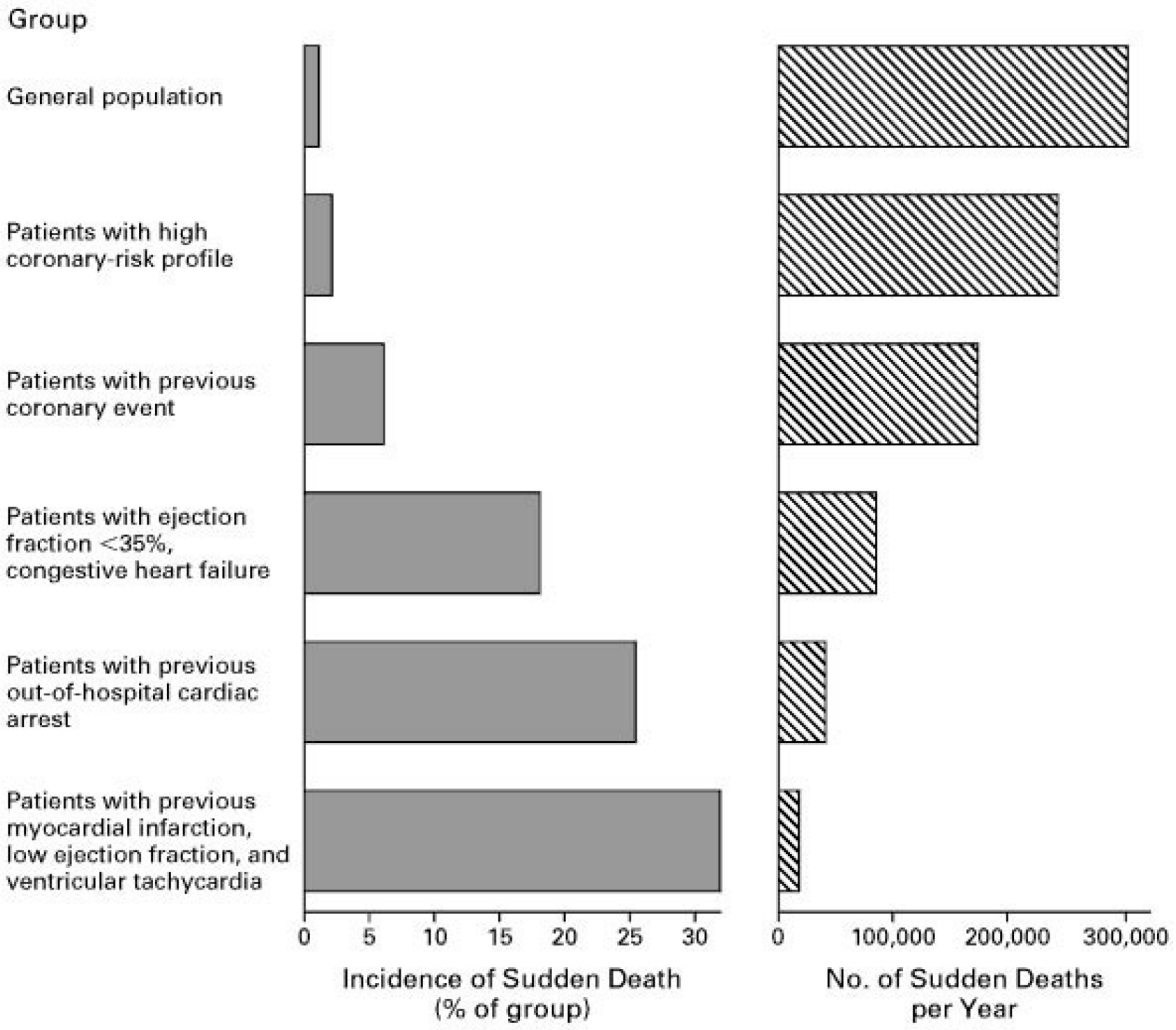
**Incidence of SCD in specific populations (reproduced from [9] with permission)**.

Worldwide, sudden cardiac death (SCD) remains a major cause of mortality, accounting for 0% to 20% of deaths in industrialized countries [1, 2]. The current annual incidence of SCD in the United States is 379,000 per year [3]. Despite a substantial reduction in age-adjusted rates of death from cardiovascular causes during the past 40 to 50 years, cardiovascular disease remains the leading natural cause of natural death in developed world. It is estimated that approximately 50 percent of all deaths from cardiovascular causes are due to SCD [4, 5]. The majority of such SCDs are caused by ventricular arrhythmias (VA), often associated with ischemic heart disease (IHD) [4, 6]. Randomized trials have shown a survival benefit of implantable cardioverter–defibrillator (ICD), compared with drug therapy, in high risk patients, particularly those with a low left ventricular ejection fraction (LVEF) and current guidelines follow these findings [7, 8]. Despite these advances, the effect on the cumulative incidence of SCD in the general population has been relatively small, because the majority of SCDs occur in patients who do not have the characteristics that would have led to their inclusion in implantable defibrillators trials (Fig. 1 (Ref. [9]): Upper 3 groups vs. 3 lower groups, respectively). Based on these trials, current guidelines provide a Class I recommendation for primary implantation of an ICD in patients with LVEF ≤35% [10]. However, with increasing left ventricular function, all-cause mortality and the absolute number of SCD increase, despite a reduction in the proportion of deaths due to cardiac arrhythmias (Fig. 1).

**Fig. 2.**
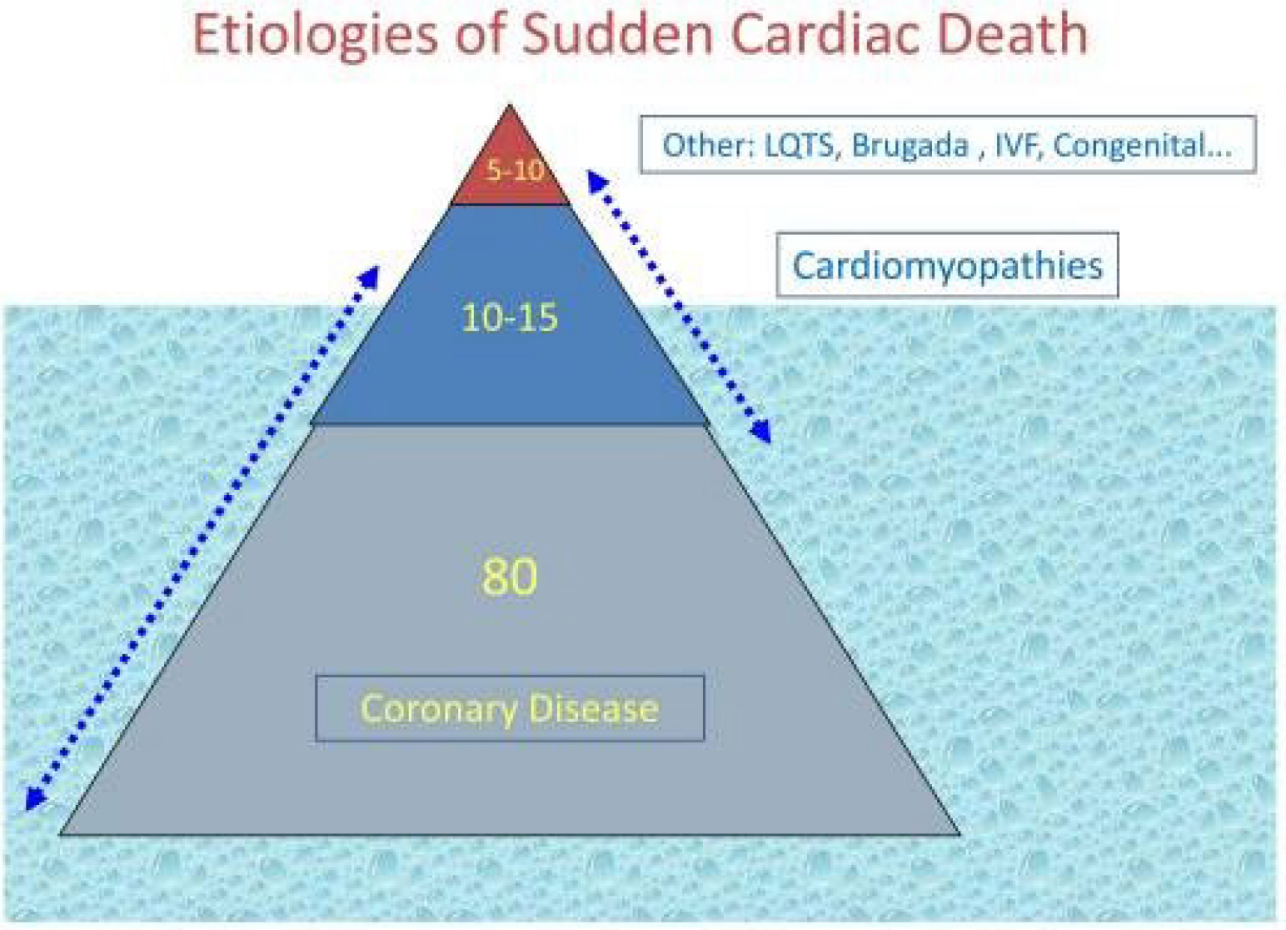
**Etiologies of SCD (reproduced from [11] with permission)**.

Ischemic heart disease (IHD), associated with obstructive coronary artery disease, accounts for approximately 80% of SCD cases (Fig. 2, Ref. [11]).

Ventricular tachycardia (VT), which initially degenerates into ventricular fibrillation (VF) and later into asystole appears to be the most common pathophysiological cascade involved in fatal arrhythmias among patients with IHD. Of note, almost half of all SCD cases in patients with IHD have normal left ventricular function and 20% have mildly or moderately decreased left ventricular systolic function (LVEF of 36% to 50%). Thus, current guidelines for primary ICD therapy are applicable to less than one third of SCD cases of IHD patients [2]. Furthermore, a low ejection fraction is an imperfect tool for clinical decision making. In various ICD trials whose inclusion criteria was based on the the EF, the Number Needed to Treat (NNT) varied. In the MADIT trial, only one third of the patients received appropriate ICD therapy for VT/VF during the trial and the rate has further declined over the years [12]. This data suggests that a more precise algorithm for risk stratification in patients with heart failure needs to be developed to have a greater impact on SCD risk reduction, using tools beyond EF.

## 2. Multifactorial Assessment of Arrhythmic Risk 

**Fig. 3.**
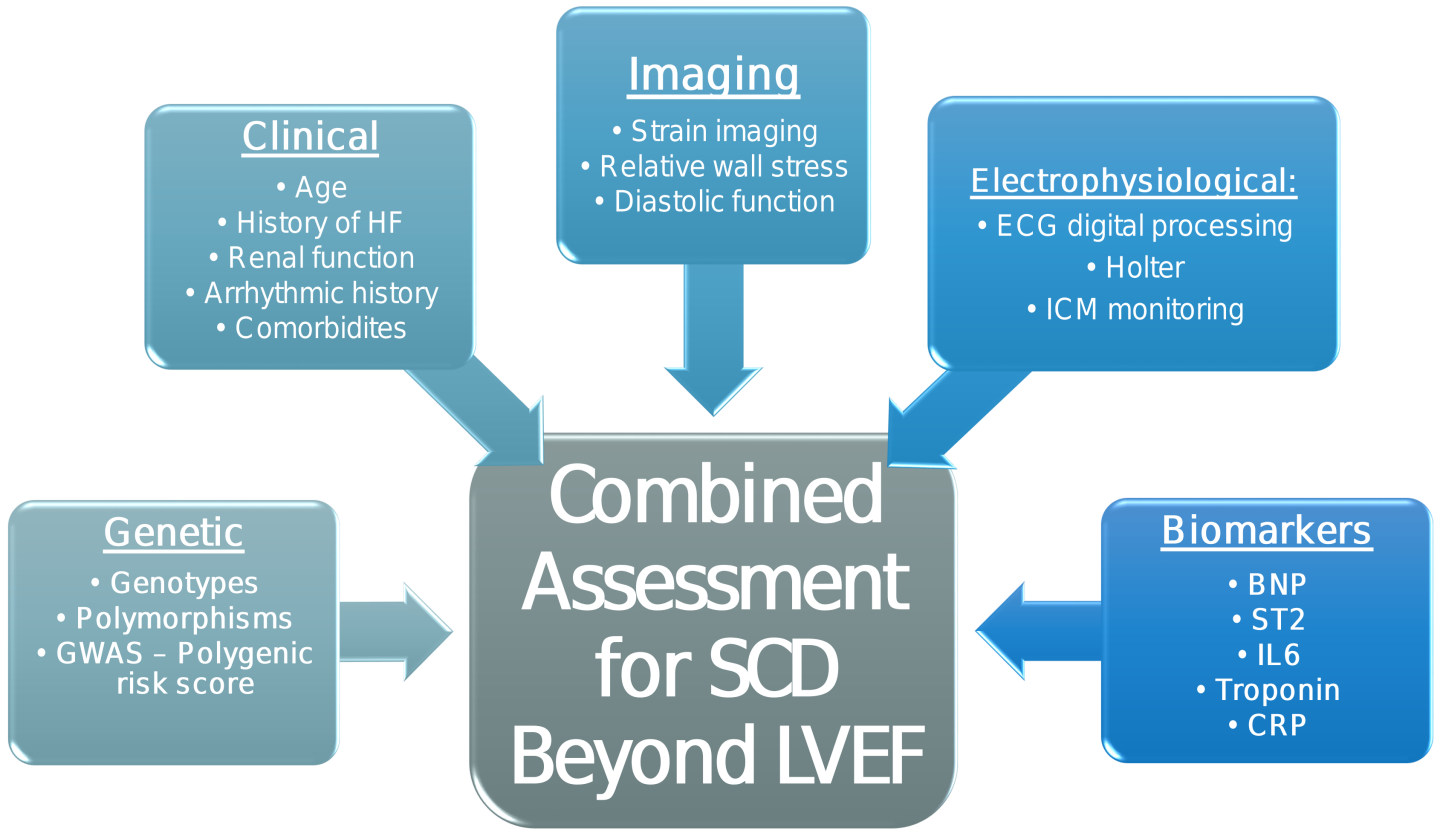
**Proposed combined risk assessment for SCD beyond LVEF**. GWAS, genome wide association study; HF, heart failure; ICM, implantable cardiac monitor; BNP, B-type natriuretic peptide; CRP, C-reactive protein.

Among all patients with IHD only 13% to 20% will have sudden cardiac arrest. Therefore, the question arises as how to identify the subgroup of patients with IHD without advanced LV dysfunction who are at the highest risk for SCD. It is likely that this risk is multifactorial, and related to clinical history and comorbidities, cardiac function and structure, pathophysiological processes related to inflammation and fibrosis, changes in electrical activation of myocytes, genetic predisposition, and possibly epigenetic alterations. Consequently, the combination of multiple risk markers in a predictive model may serve to improve the prediction of SCD risk in IHD patients beyond LVEF [13]. More advanced imaging techniques, such as echocardiographic assessment of left ventricular longitudinal strain, have been suggested to be better predictors of the development of ventricular tachyarrhythmias and SCD than LVEF alone [13] . Electrophysiological ([EP] markers have also been suggested to be important in SCD risk assessment beyond LVEF, including the presence of nonsustained ventricular arrhythmias on long-term monitoring, and ECG markers of QRS duration/morphology and of ventricular repolarization. In addition, plasma biomarkers of neurohormonal regulation, fibrosis and inflammation (such as N-Terminal pro-B type natriuretic peptide, sST2, interleukin-6 [IL6], troponin and c-reactive-protein) may also be important for SCD risk stratification [13]. Thus, studies are needed for SCD risk stratification in IHD patients without low LVEF, focusing on the combined assessment of novel modalities described above, as well as on the identification of new genetic/epigenetic markers (Fig. 3).

## 3. Advanced Analysis of ECG

**Fig. 4.**
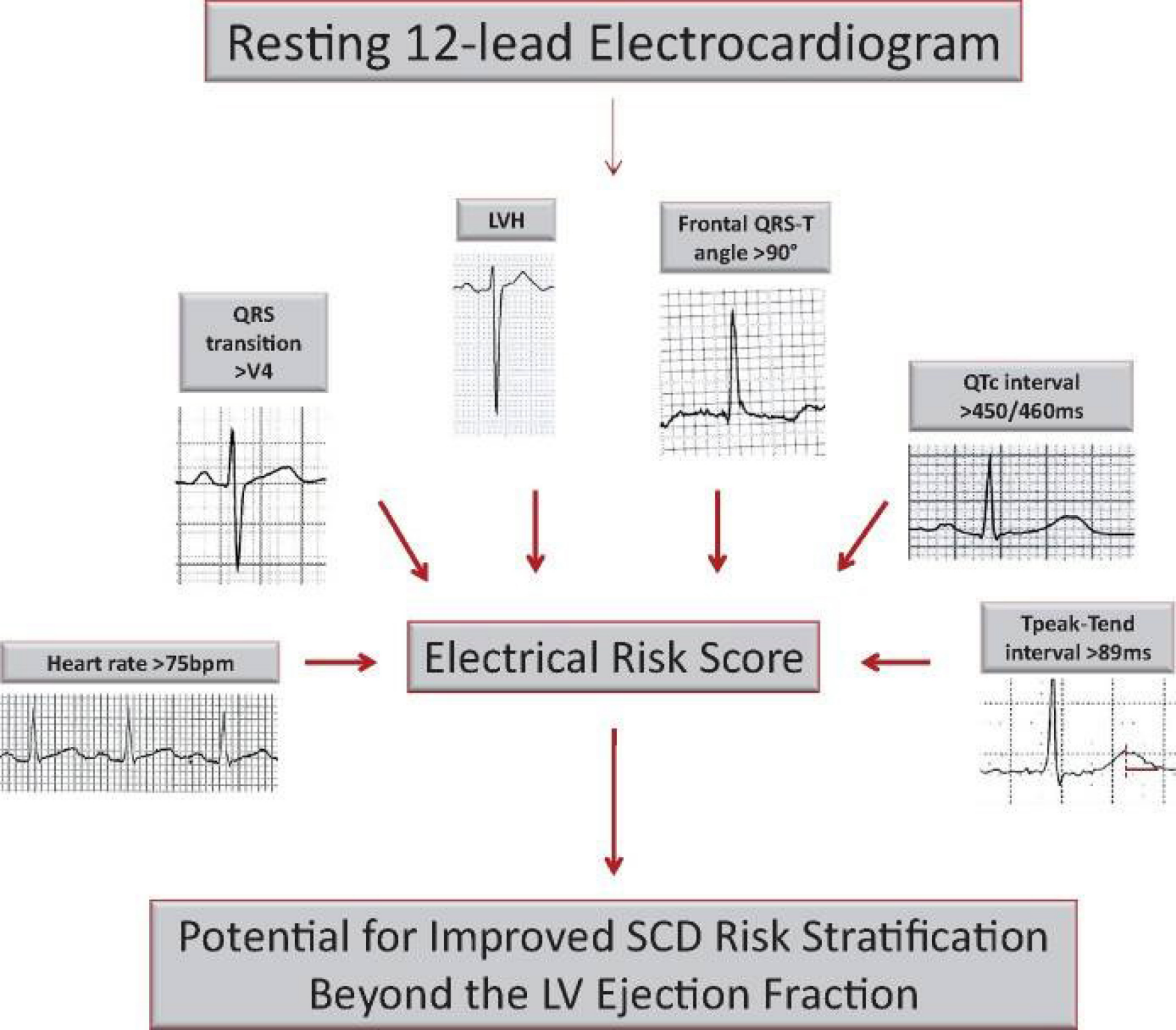
**ECG Markers of SCD Risk Beyond the LVEF (reproduced from EHJ [14] with permission)**.

The 12 lead ECG is a basic, widely available examination and use of parameters that can be derived from the ECG has been used by various investigators for prediction of sudden death. A recent report from the Oregon Sudden Unexpected Death Study (Oregon SUDS) has suggested that an ECG numeric score, consisting of a few selected parameters can lead to improved SCD risk prediction in patients with LVEF >35% (Fig. 4, Ref. [14]).

A more advanced analysis of the T wave vector may provide further risk stratification. Our group studied the value of Periodic Repolarization Dynamics (PRD), an electrocardiographic marker of sympathetic activity, as a novel approach to predict SCD in IHD patients. In an analysis of 856 post-infarction patients enrolled in the MADIT-II trial, PRD was shown to be an independent predictor of SCD (adjusted HR = 1.40; *p* = 0.003), suggesting that assessment of repolarization dynamics can be used for SCD risk stratification in IHD patients [15]. Late potentials (LPs), detecting alterations in high-frequency components within the QRS and ST segment, can identify IHD patients with increased risk for VA or SCD [16]. Further trials are ongoing to validate these scores and to employ machine learning tools to the ECG for advanced risk stratification.

## 4. Plasma Biomarkers

**Fig. 5.**
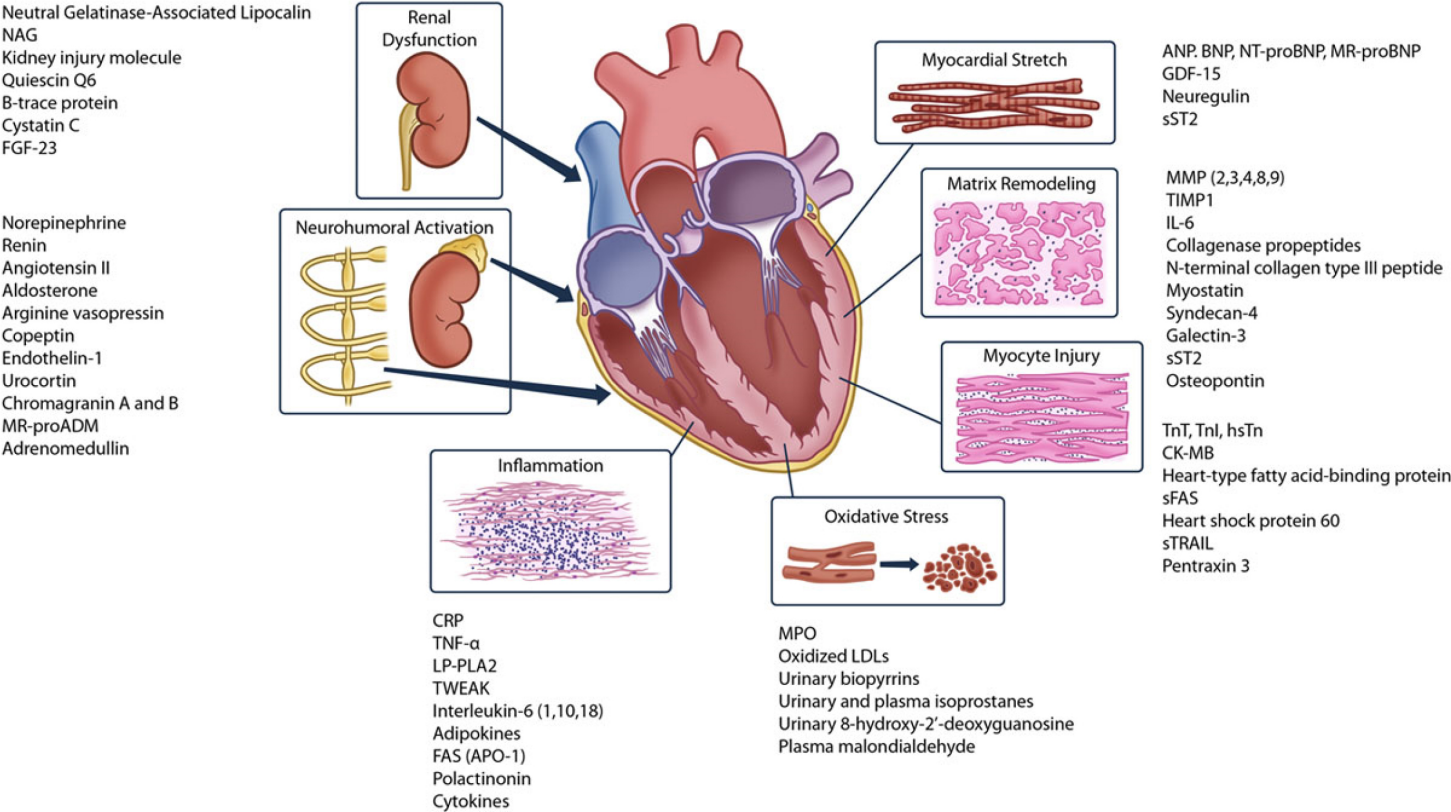
**Plasma Biomarkers for VA/SCD Risk Assessment (reproduced from [17] with permission)**. ANP, indicates atrial natriuretic peptide; APO, apolipoprotein; BNP, B-typenatriuretic peptide; CK-MB, creatinine kinase-muscle/brain; FAS, Fas cell surface death receptor; GDF, growth differentiation factor; hsTn, high-sensitivity troponin; IL, interleukin; LDL, low-density lipoprotein; LP-PLA2, lipoprotein-associated phospholipase A2; MMP, matrix metalloproteinases; MPO, myeloperoxidase; MR-proADM, midregional proadrenomedullin; MR-proBNP, midregional pro-B-type natriuretic peptide; NAG, N-acetyl β-(D)-glucosaminidase; NT-proBNP, N-terminal pro-B-type natriuretic peptide; sFAS, soluble Fas cell surface death receptor; sST2, soluble ST2; sTRAIL, soluble TNF-related apoptosis-inducing ligand; TIMP, tissue inhibitors of metalloproteinases; TNF, tumor necrosis factor; TnI, troponin I; TnT, troponin T; TWEAK, TNF-like weak inducer of apoptosis.

The intricate interplay of several mechanisms, including neurohormonal activation, inflammation, myocardial stretch, matrix remodeling, and myocyte injury, contribute to disease progression in patients with IHD (Fig. 5, Ref. [17]).

Biomarkers may provide substantial information on the complex pathophysiology that defines the syndrome of HF progression in IHD patients prior to the development of LV dysfunction and were therefore suggested to provide important prognostic information on VA risk. Natriuretic peptides are markers of neurohormonal activation and counteract the activity of the renin–angiotensin–aldosterone system and have antiproliferative and antihypertrophic effects on myocardial tissue [14, 18]. In a meta-analysis of 3453 patients without ICDs, an elevated B-type natriuretic peptide (BNP) level predicted SCD with a relative risk of 3.68 [95% CI 1.90–7.14] [19]. In MADIT-CRT, elevated baseline and follow-up BNP levels were shown to be independent predictors of increased risk for subsequent VA: in multivariate analysis elevated baseline (>median) and 1-year levels of BNP were associated with a significant increase in the risk of VT/VF (HR = 1.36; *p* = 0.02; and HR = 1.79; *p *< 0.001, respectively) [20].

**Fig. 6.**
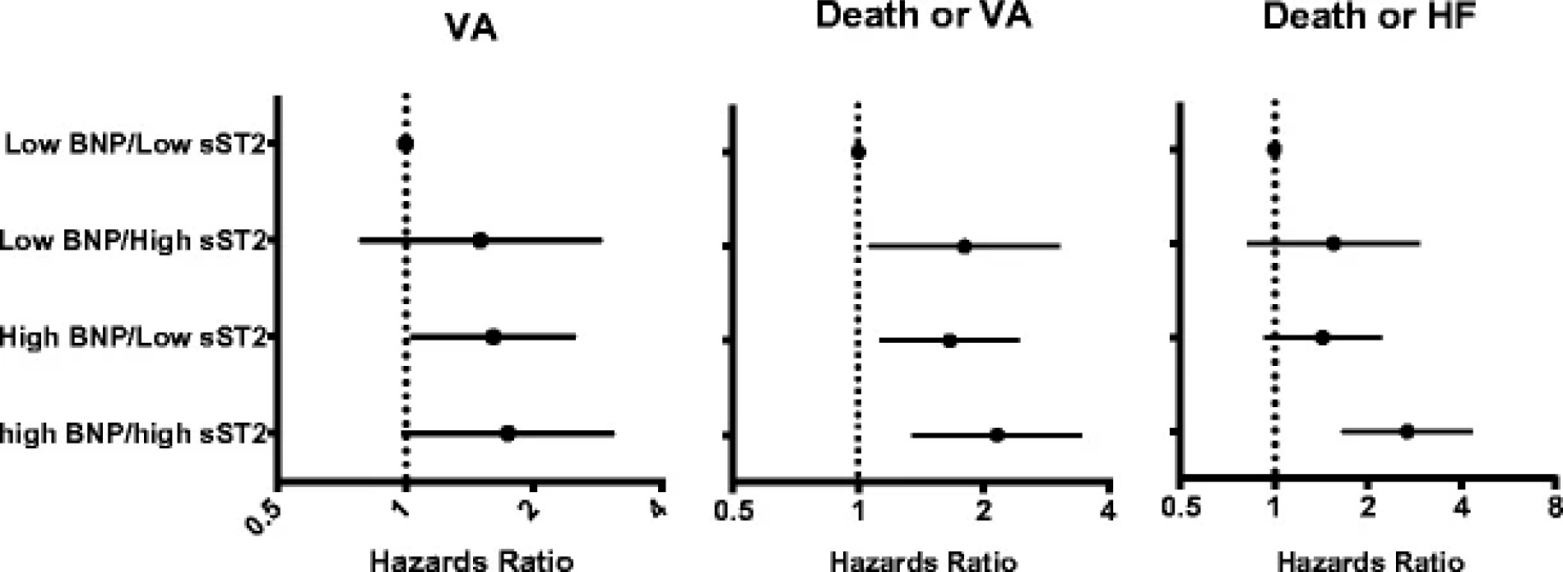
**Risk of VA, death or VA, by baseline levels of BNP and sST2 in MADIT-CRT (reproduced from [25] with permission)**.

Elevated levels of sST2 have been linked to cellular death and cardiac fibrosis [21], as well as HF disease progression, and increased risk of death [22, 23, 24]. In a cohort of 684 patients enrolled in MADIT-CRT, sST2 levels wereassessed at baseline and 1 year (n = 410). In multivariable-adjusted models, elevated baseline sST2 was associated with an increased risk of VA, even when adjusting for BNP levels at baseline (Fig. 6, Ref. [25]). Serial assessment revealed that each 10% increase in sST2 levels during follow-up was independently associated with a corresponding 11% (*p* = 0.004) increased risk of VA [25]. These data suggest that sST2 may provide incremental prognostic information for VA risk assessment.

## 5. Echocardiographic Assessment: Global Longitudinal Strain and Relative Wall Thickness

**Fig. 7.**
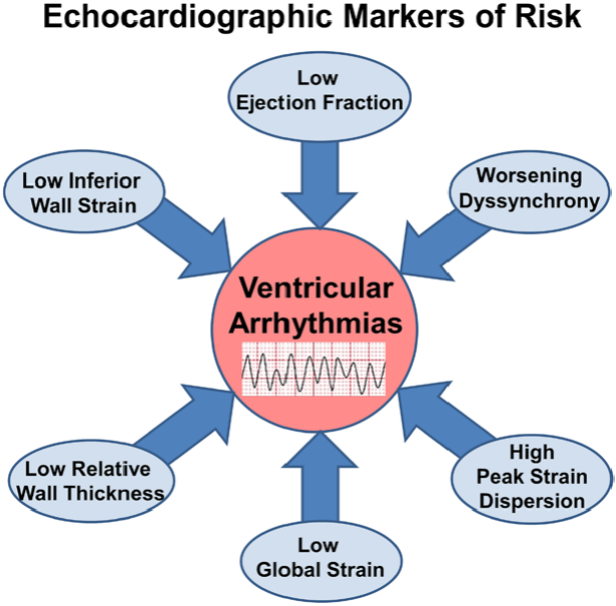
**Proposed echocardiographic markers of VA risk**.

Despite its known limitations in predicting patient risk for sudden cardiac death, EF remains the chief imaging parameter to guide ICD therapy for the primary prevention of ventricular arrhythmias. EF lacks accuracy for risk stratification and therefore other echocardiographic parameters have been proposed as risk markers of VT/VF, beyond LVEF, including low relative wall thickness [26], global longitudinal strain (GLS) [27, 28], mechanical desynchrony [29] and, more recently, peak strain dispersion [23] (Fig. 7).

**Fig. 8.**
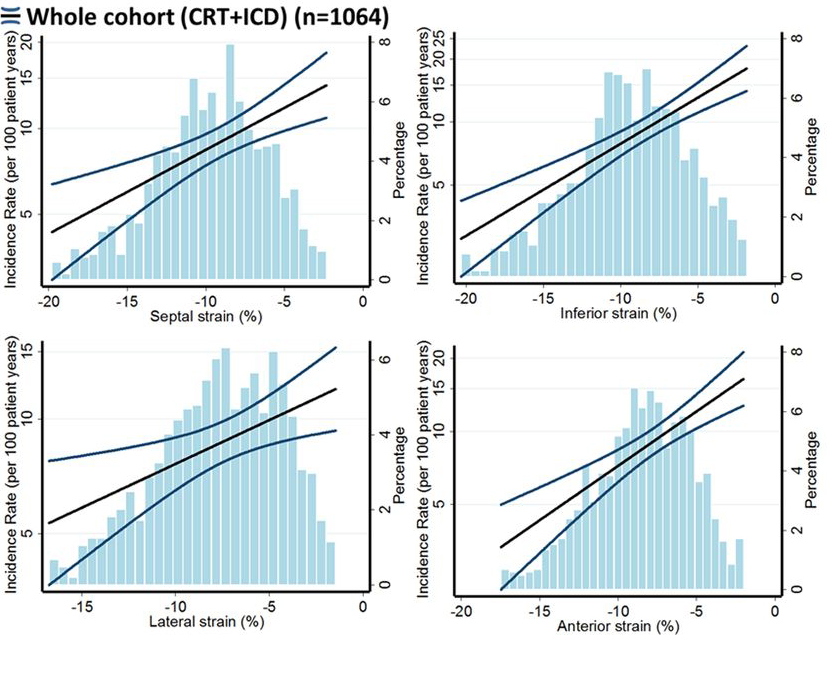
**Association of GLS (X-axis) and VA (Y-axis) in MADIT-CRT (reproduced from [30] with permission)**.

One of the most widely used parameters for prognostic utilization for strain echocardiography is GLS which is known to be more reproducible method to evaluate EF than echocardiography and has prognostic value additive to EF. Myocardial strain is a principle for quantification of LV function which is now feasible with speckle-tracking echocardiography. GLS was suggested to be more sensitive and accurate than left ventricular ejection fraction (LVEF) as a measure of systolic function, and has the advantage to detect prognostically important myocardial dysfunction even when the EF is within the normal range as a consequence it may be used to identify sub-clinical LV dysfunction in cardiomyopathies [24, 26]. The MADIT group studied 1064 patients enrolled in the MADIT-CRT trial with speckle-tracking data available [30]. Peak longitudinal strain was obtained for the septal, lateral, anterior, and inferior myocardial walls at baseline. The end point was the first occurrence of VT/VF. During the median follow-up of 2.9 years, 254 (24%) patients developed VT/VF. Patients with VT/VF had a significantly lower GLS in all myocardial walls compared with patients without VT/VF (Fig. 8, Ref. [30]), supporting the role of GLS as a marker of VA risk, independently of LVEF.

Relative wall thickness (RWT) is a measure of LV geometry suggested to be a marker for adverse events in patients without advanced LV dysfunction. It is defined as 2 times the posterior wall thickness divided by the LV diastolic diameter. Studies in hypertensive patients have shown that high RWT (concentric remodeling) is associated with increased mortality and morbidity [28, 31]. Our data from MADIT-CRT demonstrate that decreased RWT (eccentric remodeling) is associated with an increase in the risk of VA and VAdeath [26]. Compared with commonly used echocardiographic variables such as LVEF and cardiac volumes, in a multivariable model, RWT was the most powerful echocardiographic measure for estimating the risk of VAs. Patients with low RWT (<0.24) had 83% increased risk for VA and 68% increased risk in VA/death compared with patients with higher RWT values (both *p *< 0.001). Each 0.01-unit decrease in RWT was associated with 12% (*p *< 0.001) and 10% (*p *< 0.001) increases in the risk of VA and VA/death, respectively [26] .

## 6. Electrophysiological Studies

Programmed ventricular stimulation (PVS) may allow identification of those who have the substrate for sustained monomorphic ventricular arrhythmia, who may benefit from an ICD implantation. Induction of ventricular tachycardia with PVS is long knowned to identify a vulnerable substrate for SCD. A study from our group has shown that in patients with EF below 30%, inducibility at PVS will identify those who will have treatments from their ICD for VT but not for VF [32]. Thus, patients with low EF may have VA from other mechanism that are not identified by PVS. A recent study showed that ischemic patients with an EF above 40% could be risk stratified further, using clinical variables obtained from the ECG and 24 hour Holter ECG and PVS. In this study, patients that had an initial risk factor such as an prolonged QT or late potentials in the ECG, or other high risk factors per Holter such as multiple premature beats or non sustained VT, or increased T wave alternans, underwent a PVS. Those who were inducible for VT had an ICD implanted with an annual incidence of ICD discharge rate at 8.2%. The major utility of PVS is the subjects who are non-inducible, who are at low risk for ventricular arrhythmias, even in the presence of a low EF [33]. Use of PVS in non ischemic and hypertrophic cardiomyaopthies is still under investigation. The use of an invasive risk stratification strategy must be weighed against the possibility of complications of the procedure. Current guidelines recommend PVS to assist decision making in patients who have also experienced syncope [34].

## 7. Cardiac Magnetic Resonance

Cardiac magnetic resonance(CMR) imaging has been used extensively to guide risk stratification for SCD in non ischemic cardiomyopathy and hypertrophic cardiomyopathy. Several parameters of CMR have been shown to correlate with outcomes in ICM. A threshold of 15% of late gadolinium enhancement (LGE) has been shown to predict inducibility at PVS better than the EF [32, 35]. Mechanical dispersion after a first myocardial infarction correlates independently with a composite of cardiac outcomes which included death, ventricular arrhythmia [36]. Scar transmurality has been shown to be significantly associated with ICD shocks [37] . Although CMR shows great promise as a non-invasive modality for risk assessment, it still awaits prospective trials dedicated to patients with ICM especially of relatively preserved EF before it can be routinely employed to guide treatment decisions in this group.

## 8. Summary and Conclusions

Prediction of sudden cardiac death risk in the ICM patient with preserved EF accurately is a pressing need. Currently, the decision to implant an ICD for primary prevention is guided by one imperfect clinical parameter: the EF. In this review, several promising modalities were discussed that are still awaiting validation for clinical use, possibly combined with a risk scoring system. The modalities include in-depth analysis of classical tests such as features of the ECG and serum biomarkers such as pro-BNP and sST2. Advanced echocardiographic studies including longitudinal strain and RWT may enable further risk stratification. Finally, PVS and CMR which are not in routine clinical use today may add yet another critical link for risk assessment. In the future, genetic sequencing and computerized analysis using machine learning and artificial intelligence may provide the long awaited “crystal ball” of personalized medicine. In the interim, the clinician must be aware that the EF remains an imperfect tool, and judicious use of other modalities, especially in combination, may assist in shared decision making in individual patients.
